# Genomic Insights into Denitrifying Methane-Oxidizing Bacteria *Gemmobacter fulva* sp. Nov., Isolated from an *Anabaena* Culture

**DOI:** 10.3390/microorganisms9122423

**Published:** 2021-11-24

**Authors:** Long Jin, Chun-Zhi Jin, Hyung-Gwan Lee, Chang Soo Lee

**Affiliations:** 1 Co-Innovation Centre for Sustainable Forestry in Southern China, College of Biology and the Environment, Nanjing Forestry University, Nanjing 210037, China; 2 Cell Factory Research Centre, Korea Research Institute of Bioscience & Biotechnology (KRIBB), Daejeon 34141, Korea; chunsik@kribb.re.kr (C.-Z.J.); trustin@kribb.re.kr (H.-G.L.); 3 Protist Research Team, Microbial Research Department, Nakdonggang National Institute of Biological Resources, Sangju 37242, Korea

**Keywords:** *Gemmobacter*, *Gemmobacter fulva*, denitrification, methane oxidation

## Abstract

The genus *Gemmobacter* grows phototrophically, aerobically, or anaerobically, and utilizes methylated amine. Here, we present two high-quality complete genomes of the strains con4 and con5^T^ isolated from a culture of *Anabaena*. The strains possess sMMO (soluble methane monooxygenase)-oxidizing alkanes to carbon dioxide. Functional genes for methane-oxidation (*prmAC*, *mimBD*, *adh*, *gfa*, *fdh*) were identified. The genome of strain con5^T^ contains *nirB*, *nirK*, *nirQ*, *norB*, *norC*, and *norG* genes involved in dissimilatory nitrate reduction. The presence of nitrite reductase gene (*nirK*) and the nitric-oxide reductase gene (*norB*) indicates that it could potentially use nitrite as an electron acceptor in anoxic environments. Taxonomic investigations were also performed on two strains through polyphasic methods, proposing two isolates as a novel species of the genus *Gemmobacter*. The findings obtained through the whole genome analyses provide genome-based evidence of complete oxidation of methane to carbon dioxide. This study provides a genetic blueprint of *Gemmobacter* *fulva* con5^T^ and its biochemical characteristics, which help us to understand the evolutionary biology of the genus *Gemmobacter*.

## 1. Introduction

*Gemmobacter* is of interest because of its metabolic pathways and its habitats. Members of the *Gemmobacter* species are able to grow phototrophically, aerobically, and anaerobically [1,2,3,4,5,6]. The first species, *Gemmobacter changlensis*, isolated from a snow sample collected in the Indian Himalayas, was proposed as psychrotolerant and phototrophic bacteria [1]. Another phototrophically growing species, *Gemmobacter aestuarii*, recovered from estuarine surface water, was described as containing a complete gene cluster for photosynthesis [3]. The members of the genus *Gemmobacter* have been discovered in a wide range of natural environments, such as freshwater, sulphuric cave waters, estuaries, forest ponds, artificial fountains, tidal flats, activated sludge, a white stork nestling, coastal planktonic seaweed, and conserved forages, indicating that members of this genus are widely distributed in natural and artificial environments [2,4,5,6,7,8,9,10,11,12,13,14,15].

Comparative genomics analyses revealed that members of the genus *Gemmobacter*, including *Gemmobacter aquatilis*, *Gemmobacter lutimaris*, *Gemmobacter* sp. HYN0069, *Gemmobacter caeni*, and *Gemmobacter* sp. LW-1, utilize methylated amine. These species have related genes encoding the enzymes trimethylamine (TMA) dehydrogenase, TMA monooxygenase, and TMA demethylase in their genome, indicating metabolic potential of using the TMA oxidation pathway to convert trimethylamine to dimethylamine [3,8]. In a recent study, we isolated two alphaproteobacterial strains, con4 and con5^T^, from an *Anabaena* culture that contained all genes related to the oxidization of methane to carbon dioxide.

The species in the genus *Gemmobacter* were characterized as Gram-negative, oxidase and catalase-positive, non-spore-forming, and rod-shaped containing ubiquinone-10 as the major respiratory quinone, with G + C content in the range of 61.4–69.1 mol% [1,3,4,5]. Here, we report a comparative analysis of the genomes of two strains, con4 and con5^T^, together with a taxonomic proposal based on their phylogenetic, genomic, physiological, and chemotaxonomic characteristics.

## 2. Material and Methods

### 2.1. Isolation and Culture Conditions of the Strains

Strains con5^T^ and con4 were recovered from a culture of *Anabaena* using a serial dilution method [16]. For the *Anabaena* culture, *Anabaena variabilis* FBCC010004 strain was obtained from the Freshwater Bioresources Culture Collection (FBCC, https://fbcc.nibr.re.kr/fbcc, accessed on 25 August 2021) of the Nakdonggang National Institute of Biological Resources (NNIBR, South Korea). *Anabaena* cells were cultured in 500 mL standard cell culture flasks with Blue–Green (BG11) broth (Merck, St. Louis, MO, USA) under the conditions: 20 °C, 35% humidity, 12 h:12 h light-dark photoperiod, 20 μmol m^−2^ s^−1^ irradiance, and 200 rpm agitation [17]. From the *Anabaena* culture, a 100 μL sub-sample of the suspended material was aseptically spread onto modified R2A agar (Difco, NJ, USA) [18] and incubated at 25 °C under heterotrophic conditions. Two yellow-pigmented strains, con5^T^ and con4, were isolated after three days and routinely sub-cultivated on R2A agar at 30 °C for 48 h and kept in a glycerol solution (20%, *v*/*v*) at −70 °C for long-term preservation. For most experiments, all strains were cultivated on R2A agar at 30 °C.

### 2.2. Morphological, Physiological, and Chemotaxonomic Characteristics

Cell motility and morphology were examined via a phase-contrast microscope (Nikon Optiphot, 1000 × magnification) and transmission electron microscopy (Philips CM-20) using cells grown for 48 h in R2A media at 30 °C. The growth temperature was tested at 4, 8, 15, 20, 30, 37, and 45 °C. The pH range for growth was determined using different buffering systems: citrate–phosphate for pH 4–7, phosphate for pH 8, and glycine–NaOH for pH 9–11 [19]. Salt tolerance was checked on NaCl-free R2A agar with different NaCl concentrations 1.0–5.0% (*w*/*v*; at intervals of 1.0%) NaCl. Anaerobic growth was examined after incubating strains con4 and con5^T^ on R2A agar in an anaerobic glove box (Coy Laboratory Products, Grass Lake, MI, USA) containing an atmosphere of 99% N_2_ and 1% H_2_ and equipped with palladium catalyst packs to remove oxygen and a gas analyzer to monitor oxygen concentrations. Cell growth at different media was investigated in different media of R2A agar, tryptic soy agar (TSA; Difco, NJ, USA), nutrient agar (NA; Difco, NJ, USA), and Luria–Bertani (LB; Difco, NJ, USA) medium. The antimicrobial susceptibility of the strains was checked on R2A agar medium using the filter-paper disk method [20], where the disks contained the following: amikacin (30 µg mL^−1^), ampicillin/sulbactam (20 µg mL^−1^, 1:1), chloramphenicol (30 µg mL^−1^), erythromycin (30 µg mL^−1^), gentamicin (30 µg mL^−1^), kanamycin (30 µg mL^−1^), lincomycin (15 µg mL^−1^), nalidixic acid (30 µg mL^−1^), rifampicin (30 µg mL^−1^), spectinomycin (25 µg mL^−1^), streptomycin (25 µg mL^−1^), teicoplanin (30 µg mL^−1^), tetracycline (30 µg mL^−1^), and vancomycin (30 µg mL^−1^). The haloes for susceptibility were recorded when the diameters were more than 10 mm after incubation at 30 °C for 2 days. Other biochemical activities were examined using API 20NE, API ID 32GN, and API ZYM kits (bioMérieux, l’Etoile, France) according to the manufacturer’s instructions.

The whole-cell fatty acid profile was analyzed by gas chromatography (Hewlett Packard 6890, Kyoto, Japan) with the TSBA 6 database using Sherlock software v6.1 at KCTC (Korean Collection for Type Cultures) service center. The cells of the strains con5^T^ and con4 and four *Gemmobacter*-type strains were collected after being grown at 30 °C for 48 h on the same sectors of R2A agar plates. Cell-harvesting standardization was done following the method described by Jin et al. [18]. The extraction of respiratory quinones and polar lipids was completed following the method described previously [21,22]. The respiratory quinones were identified using HPLC (Shimadzu, Kyoto, Japan) with an YMC-Pack ODS-A column, and the polar lipids were identified with two-dimensional TLC plates (60 F_254_, Merck).

### 2.3. Phylogenetic and Genomic Analyses

For a 16S rRNA gene analysis, genomic DNA of strains con5^T^ and con4 was extracted using a FastDNA^TM^ SPIN kit. Amplification of the 16S rRNA genes was done by PCR with 27F/1492R primer sets [23]. The 16S rRNA gene sequences of strains con5^T^ and con4 were compared with the available sequences in the EzBioCloud server [24]. Evolutionary analyses were performed with mega7 software [25]. Sequence edition and multiple alignment were done using the programs bioedit [26] and clustal x [27], respectively. Phylogenetic trees were reconstructed using three algorithms: Neighbor–Joining (NJ), Maximum–Parsimony (MP), and Maximum–Likelihood (ML) [28,29,30]. The ranch robustness of the phylogenetic trees was estimated by bootstrap analyses based on 1000 re-samplings of the sequences [31]. A phylogenomic tree was re-constructed from the available type strains of species of the genus *Gemmobacter* with whole genome sequences on the TYGS (Type Strain Genome Server) [32]. The phylogenomic tree was inferred using FastME 2.1.4 [33] from GBDP (Genome Blast Distance Phylogeny) distances calculated from genome sequences. Branch lengths were calculated using the GBDP distance formula d5 [34]. The whole genomes of strains con5^T^ and con4 were sequenced using both the PacBio RSII platform and Illumina next-generation sequencing technology at Macrogen Inc. (Seoul, South Korea). The chromosomes and plasmids were assembled using the software package of SMRT portal (v.3.2.0), Pacific Biosciences, (CA, USA) [35]. The de novo genome annotation of strains con5^T^ and con4 was performed with the Prokka (v.1.4.6) pipeline [36], and a sequence-based comparison was made using the SEED Viewer [37]. The predicted coding sequences (CDSs) were submitted to the COG database to create the functional categories [38,39]. The values of average nucleotide identity (ANI) were calculated by using the OrthoANI tool in EZBioCloud server [40], and the digital DNA–DNA hybridization (dDDH) values were calculated using the genome-to-genome distance calculator (GGDC 2.1) based on draft genome sequences [34].

## 3. Results and Discussion

### 3.1. Physiological Tests

The two strains con5^T^ and con4 were Gram-negative, non-motile, aerobic, and rod-shaped (Supplementary Figure S1). The colonies appeared yellow, convex, circular, and smooth, with entire edges, after being grown for two days at 30 °C on R2A agar. Cell growth was observed at temperatures ranging from 4 to 37 °C and at pH 5–10 (weak at pH 5). Oxidase and catalase activities were present. The cells were found to assimilate *N*-acetyl-glucosamine, d-glucose, d-mannitol, d-mannose, malate, and maltose but not adipate, l-arabinose, caprate, citrate, gluconate, and phenyl acetate (API 20NE). The cells were found to be positive for the following enzyme activities (API ZYM test strip): esterase (C4), esterase lipase (C8), *α*-glucosidase, leucine arylamidase, and naphthol-AS-BI-phosphohydrolase. However, the cells were found to be negative for *N*-acetyl-*β*-glucosaminidase, acid phosphatase, alkaline phosphatase, *α*-chymotrypsin, cystine arylamidase, *α*-fucosidase, *α*-galactosidase, *β*-galactosidase, *β*-glucosidase, *β*-glucuronidase, lipase (C14), *α*-mannosidase, trypsin, and valine arylamidase (Table 1). The cells were found to be susceptible to amikacin (30 µg mL^−1^), ampicillin/sulbactam (1:1; µg mL^−1^), chloramphenicol (30 µg mL^−1^), erythromycin (30 µg mL^−1^), gentamicin (30 µg mL^−1^), kanamycin (30 µg mL^−1^), nalidixic acid (30 µg mL^−1^), rifampicin (30 µg mL^−1^), spectinomycin (25 µg mL^−1^), streptomycin (25 µg mL^−1^), teicoplanin (30 µg mL^−1^), tetracycline (30 µg mL^−1^), and vancomycin (30 µg mL^−1^) but resistant to lincomycin (15 µg mL^−1^).

The major fatty acids were summed feature 8 (comprising C_18:1_ *ω*7*c* and/or C_18:1_ *ω*6*c*) for both strains (data not shown). The major fatty acids in strains con5^T^ and con4 were consistent with the major fatty acid components in species from the genus *Gemmobacter*. Notably, con5^T^ and con4 differed from the four closest relatives in the proportions of some minor fatty acids. The major predominant respiratory ubiquinone was Q-10. The polar lipids consisted of phosphatidylethanolamine (PE), phosphatidylglycerol (PG), phosphatidylcholine (PC), two unidentified glycolipids (GL1, GL2), three phospholipids (PL1, PL2, and PL3), and three unidentified aminophospholipids (APL1, APL2, and APL3) for the type strain con5^T^; and phosphatidylethanolamine (PE), phosphatidylglycerol (PG), phosphatidylcholine (PC), two unidentified glycolipids (GL1, GL2), three unidentified phospholipids (PL1, PL2, and PL3), and two unidentified aminophospholipids (APL1, APL2) for the strain con4 (Supplementary Figure S2). This profile is similar to that of closely related species *G. changlensis* and *G*. *aquaticus*, with major components of PE, PG, and PC, and strains con5^T^ and con4 contain two unidentified glycolipids that differentiate these two novel strains from close members in the genus *Gemmobacter*.

### 3.2. Phylogenetic and Genomic Analysis: The Taxonomic Status

**Figure 1:**
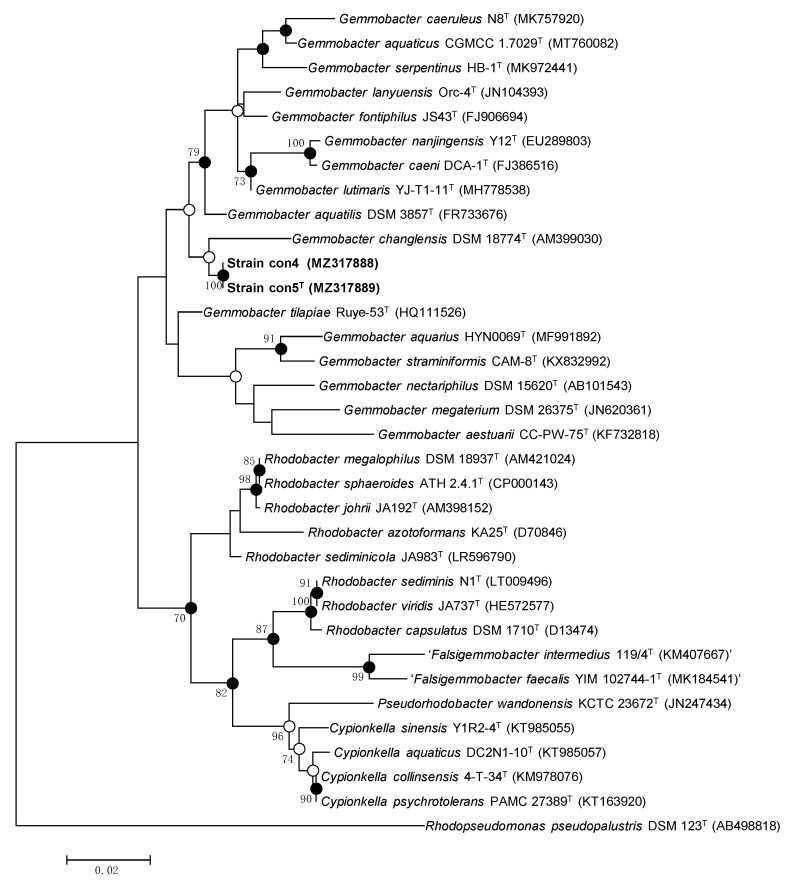
Maximum likelihood phylogenetic tree based on 16S rRNA gene sequences showing the position of strains con5^T^ and con4 within *Gemmobacter*. Sequences of related species in the *Rhodobacteraceae* family are shown. The numbers at the branch-nodes represent the percentage of 1000 bootstrap replicates; only values above 70% are depicted in the tree. Filled circles indicate the corresponding nodes recovered by ML, MP, and NJ phylogenetic trees. Open circles indicate the corresponding nodes were also recovered with either the ML or MP algorithm. The analysis involved a total of 1308 aligned nucleotide positions in the final dataset.

The 16S rRNA sequences of strains con5^T^ and con4 share 100% identity between them and 94.9–97.9% identity with those of the closest species within the genus *Gemmobacter* (Table 2). The 16S rRNA gene sequences of strains con5^T^ and con4 were compared with the 16S rRNA gene sequences of representative species within the genus *Gemmobacter* and related genera in the EzTaxon-e server. The two strains share over 97.0% similarity with *G. aquaticus* A1-9^T^ (97.9%), *G. caeruleus* N8^T^ (97.7%), *G. lutimaris* YJ-T1-11^T^ (97.4%), and *G. tilapiae* KCTC 23310^T^ (97.3%) and less than 97% with the remaining species within the genus *Gemmobacter*. Strains con5^T^ and con4 also share high similarities with other species than members of *Gemmobacter*: 96.7% with *Cypionkella collinsensis* 4-T-34^T^, 96.7% with *Cypionkella psychrotolerans* PAMC 27389^T^, and 96.4% with *Cypionkella aquatica* DC2N1-10^T^. However, it was clear from the topology of the phylogenetic tree (Figure 1) that strains con5^T^ and con4 clustered clearly with the species of *Gemmobacter*. In addition, the phylogenomic tree reconstructed on the TYGS provided clearer evidence for the taxonomic position of the two strains within the genus *Gemmobacter* (Supplementary Figure S3). The genomic DNA G + C content of the two strains was 64.1 mol%, which is in the range reported previously for the genus *Gemmobacter* (61.4–69.4 mol%) [3,4]. The ANI and dDDH values of strains con5^T^ and con4 with other available type strains of *Gemmobacter* were 73.31–80.61 % and 19.03–23.15 % (Table 2, Supplementary Figure S4), respectively, which were much lower than the species boundaries of ANI or dDDH of 95–96% and 70%, respectively, and fall in the intergeneric range [41,42,43].

### 3.3. Genome Properties

The genome of strain con5^T^ was 4.7 Mb and contained a circular chromosome of 3.4 Mb and six plasmids sized 34.0–425.5 kb (CP076361–CP076367) (Supplementary Tables S1 and S2). Of 4534 genes, 4472 were protein-coding genes and 62 were RNA genes (nine rRNA genes, 52 tRNA genes, and one tmRNA gene). For strain con4 (JAHHWR000000000), of 4417 genes, 4317 were protein-coding genes and 53 were RNA genes (four rRNA genes, 46 tRNA genes, one tmRNA gene, and two ncRNA genes) (Supplementary Figure S5). The DNA G + C content of both strains was 64.1 mol% (Supplementary Tables S1 and S2).

### 3.4. Genome Analyses for Denitrification and Methane Oxidation

It is generally understood that methanotrophic bacteria are mostly active at the oxic-anoxic transition zone in stratified lakes, using oxygen to oxidize methane. The methanotrophs produce methane monooxygenase to utilize methane as a carbon source [44,45]. The methanotrophs express two kinds of methane monooxygenase, soluble methane monooxygenase (sMMO) and particulate methane monooxygenase (pMMO), and these enzymes can also oxidize various alkanes [46,47,48].

**Figure 2:**
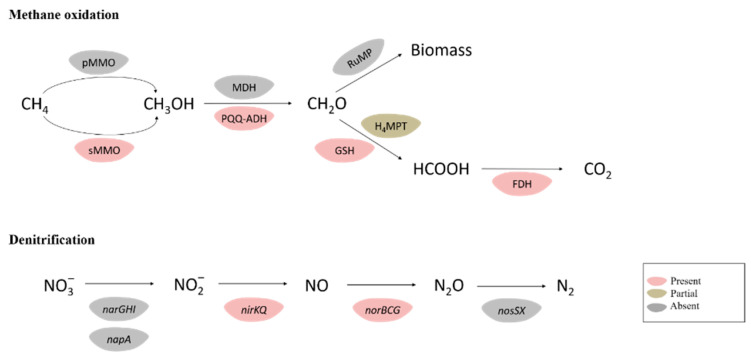
Metabolic pathways of methane oxidation and denitrification in the strain con5^T^. Enzymes and pathways indicated in light-pink were encoded by the genome; light-green indicates the presence of an incomplete pathway. Grey pathways and genes were not detected. FDH, formate dehydrogenase; GSH, glutathione (GSH)-dependent pathway; H4F, methylene tetrahydrofolate pathway; H4MPT, tetrahydromethanopterin pathway; MDH, methanol dehydrogenase; PQQ-ADH, PQQ-dependent alcohol dehydrogenase; RuMP, ribulose monophosphate pathway.

**Figure 3:**
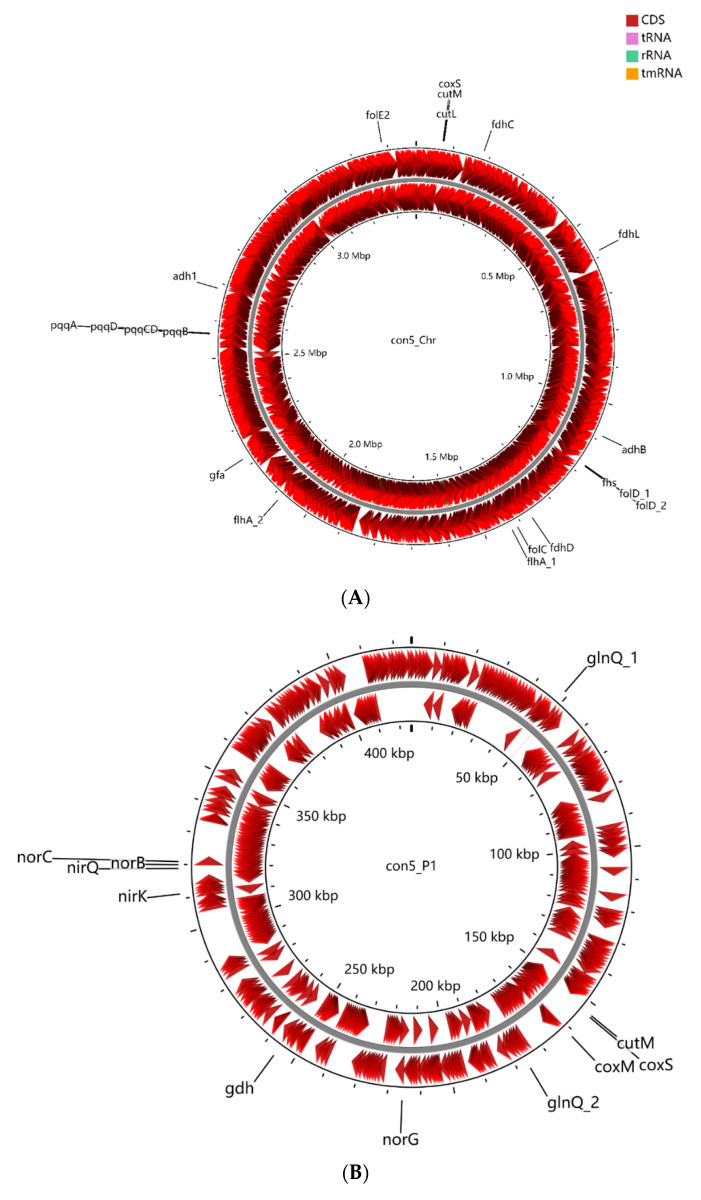

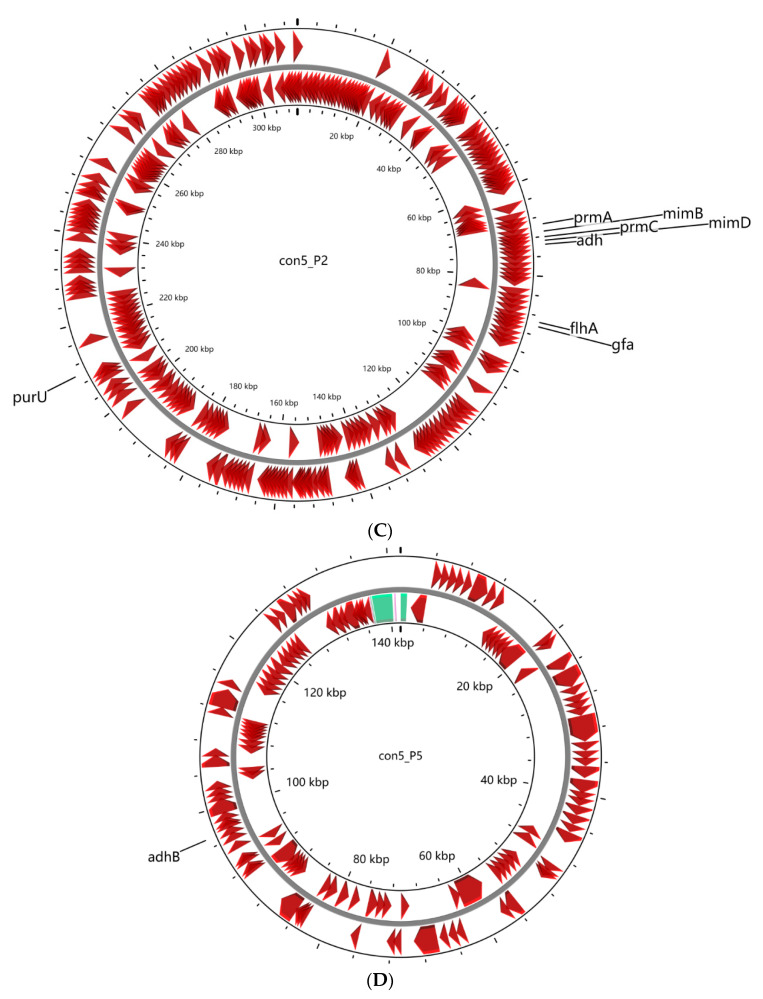
Graphic representation of circular chromosome (**A**) and plasmids (**B**–**D**) of strain con5^T^. The locations of genes involved in methylotrophy are indicated at the outside of the map. The locations of genes involved in methylotrophy are indicated at the outside of the map. The CDS (dark red), tRNA (purple), rRNA (green), and tmRNA (golden) were shown on the reverse and forward strand, respectively.

**Figure 4:**
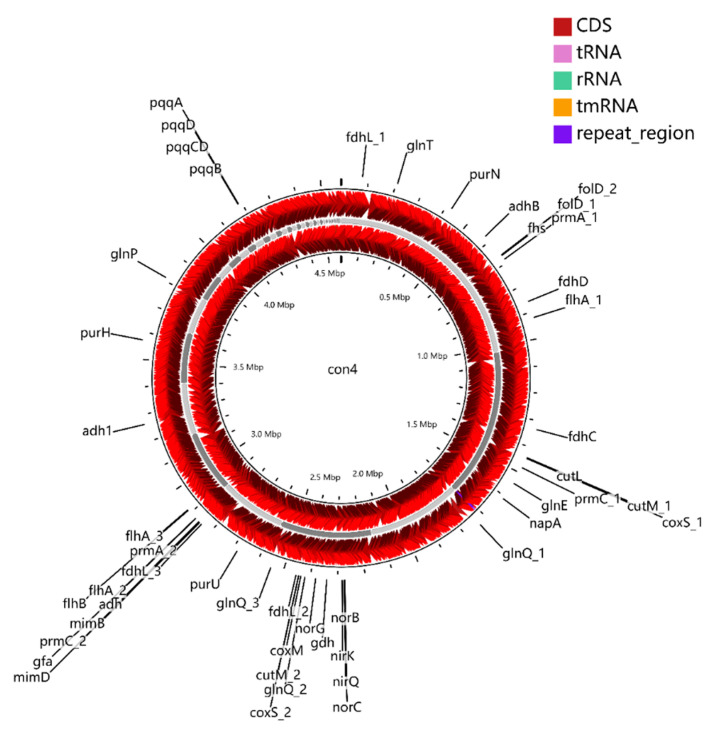
Graphic representation of circular genome of strain con4. The locations of genes involved in methylotrophy are indicated at the outside of the map. The locations of genes involved in methylotrophy are indicated at the outside of the map. The CDS (dark red), tRNA (purple), rRNA (green), and tmRNA (golden) were shown on the reverse and forward strand, respectively.

Strain con5^T^ possesses sMMO (*prmAC, mimBD*) but does not have a pMMO gene in its genome sequence. The presence of one PQQ-dependent alcohol dehydrogenase (ADH) (PQQ-MDH: *adh*, *adh1,* and *adhB*), two NAD^+^-dependent ADH (*adh*, *adh1*), and another alcohol dehydrogenase (*adhB*) in strain con5^T^ makes it a good candidate for the conversion of methane to aldehyde (Figure 2). Genes involved in the glutathione (GSH)-dependent pathway to metabolize formaldehyde are identified. Formaldehyde (HCHO)-activating enzyme (*gfa*), a GSH-dependent formaldehyde dehydrogenase (*fdh*), and *S*-formyl-GSH hydrolase (*fgh*) convert formaldehyde to formate, and then the formate is oxidized by formate dehydrogenase (*fdh*) to the final product, carbon dioxide (CO_2_) (Figure 2 and Figure 3). Those genes were also found in the strain con4 (Figure 4).

Some methanotrophs comprise genes encoding enzymes for the nitrate reduction pathway, which was confirmed to be related to methane oxidation under anoxic conditions [49], and these methanotrophic species encode a complete nitrate reduction pathway to use nitrate as a terminal electron acceptor when oxygen is depleted [50]. The genome of strain con5^T^ contains *nirB*, *nirK*, *nirQ*, *norB*, *norC*, and *norG* genes involved in dissimilatory nitrate reduction, but no dissimilatory nitrate reductase (*narG*) or nitrous oxide reductase (*nosZ*) genes are detected, which is incomplete for the denitrification pathway (Figure 2 and Figure 3). This suggests that strain con5^T^ cannot perform nitrate reduction, itself, unless it utilizes unknown or incorrectly classified reduction pathways up to date. The genome of strain con5^T^ contains the nitrite reductase gene, *nirK*, and the nitric-oxide reductase gene *norB*, and it could potentially use nitrite as an electron acceptor in anoxic environments.

## 4. Conclusions

In this study, the strains con5^T^ and con4, representing methane oxidizing species from an *Anabaena* culture belonging to the genus *Gemmobacter*, were investigated using genomic and polyphasic methods. The findings obtained through the whole genome analyses provide genome-based evidence of complete oxidation of methane to carbon dioxide. This study provides a genetic blueprint of *Gemmobacter fulva* con5^T^ and its biochemical characteristics, which help us to understand the evolutionary biology of the genus *Gemmobacter*. Based on the phylogenetic position and the genotypic, chemotaxonomic, and physiological differences, we propose that strains con5^T^ and con4, *Gemmobacter fulva* sp. nov., should be assigned as a novel species of the genus *Gemmobacter* in the family *Rhodobacteraceae* (Table 3).

## 5. Nucleotide Sequence Accession Numbers

The GenBank/EMBL/DDBJ accession numbers for the 16S rRNA gene sequences of strains con5^T^ and con4 are MZ317888 and MZ317889, respectively. Accession numbers for the genome sequences of strains con5^T^ and con4 are CP076361–CP076367 and JAHHWR000000000, respectively.

## Figures and Tables

**Table 1:** Features that differentiate strains con5^T^ and con4 from type strains of recognized *Gemmobacter* species: Strains: 1, con5^T^; 2, con4; 3, *G. changlensis* KCTC 5728^T^; 4, *G. aquatilis* DSM 3857^T^; 5, *G. lutimaris* KCTC 62715^T^; 6, *G. tilapiae* KCTC 23310^T^. All data are from this study unless indicated. +, positive; −, negative; nd, not determined.

Characteristics	1	2	3	4	5	6
Isolation source	Anabaena culture	Anabaena culture	snow sample	forest pond	tidal flat	freshwater
Colony color	yellow	yellow	yellowish brown	colorless	cream	creamy white
NaCl tolerance range (*w*/*v*%)	0–2.0	0	0–4.0	0–2.0	0–7.0	0–1.0
Indole production	−	−	−	+	−	−
Gelatin hydrolysis	+	+	−	−	+	−
Carbon utilization:						
*N*-Acetyl-glucosamine	+	+	+	−	+	−
Adipate	−	−	−	−	−	−
l-Arabinose	−	−	+	−	+	−
Caprate	−	−	−	−	−	−
Citrate	−	−	−	−	+	−
Gluconate	−	−	+	−	−	−
d-Glucose	+	+	+	−	+	+
Malate	+	+	+	−	+	−
Maltose	+	+	+	−	+	−
d-Mannitol	+	+	+	−	+	+
d-Mannose	+	+	+	−	+	−
Phenyl acetate	−	−	−	−	+	−
Enzyme activity:						
*N*-Acetyl-*β*-glucosaminidase	−	−	−	−	−	+
Acid phosphatase	−	−	+	+	+	+
Alkaline phosphatase	−	−	+	+	+	+
Cystine arylamidase	−	−	−	+	−	+
*β*-Galactosidase	−	−	−	+	+	+
*β*-Glucosidase	−	−	−	+	−	+
Lipase (C14)	−	−	−	+	−	+
Valine arylamidase	−	−	−	+	−	+
Major polar lipids	PG, PE, PC, GL	PG, PE, PC, GL	PG, PE, PC, GL, AL	PG, PE, PC, PL	PG, PE, PC, L	PG, PE, PC, AL
DNA G + C content (mol%)	64.1	64.1	69.1	65.1	65.6	61.5

**Table 2:** General features and relationship of the genomes of strains con5^T^ and con4 with the closely related species of the genus *Gemmobacter*.

No.	Strains	1. con5^T^ (%)			2. con4 (%)		
		16S rDNA	ANI	dDDH	16S rDNA	ANI	dDDH
1	*Gemmobacter* sp. con5^T^ (CP076361-CP076367)	–	–	–	–	–	–
2	*Gemmobacter* sp. con4 (JAHHWR000000000)	100	99.99	98.29	–	–	–
3	*Gemmobacter aestuarii* CC-PW-75^T^ (SSND00000000)	94.9	75.85	22.62	94.9	75.85	22.67
4	*Gemmobacter aquaticus* A1-9^T^ (VOAK00000000)	96.4	76.46	21.81	96.4	76.45	21.86
5	*Gemmobacter aquatilis* DSM 3857^T^ (FOCE00000000)	97.9	80.49	19.03	97.9	80.61	19.07
6	*Gemmobacter caeni* CGMCC 1.7745^T^ (VLLH00000000)	96.4	78.22	20.76	96.4	78.16	20.89
7	*Gemmobacter caeruleus* N8^T^ (VKKX00000000)	96.0	78.59	20.61	96.0	78.62	20.64
8	*Gemmobacter changlensis* JA139^T^ (PZKG00000000)	97.7	75.84	21.99	97.7	75.79	22.07
9	*Gemmobacter lanyuensis* KCTC 23714^T^ (BMYQ00000000)	96.2	77.76	21.35	96.2	77.69	21.39
10	*Gemmobacter lutimaris* YJ-T1-11^T^ (QXXQ00000000)	97.4	78.57	20.54	97.4	78.42	20.68
11	*Gemmobacter megaterium* DSM 26375^T^ (FTOT00000000)	95.6	73.82	22.49	95.6	73.76	22.59
12	*Gemmobacter nanjingensis* KCTC 23298^T^ (BMYI00000000)	95.8	78.16	20.68	95.8	78.31	20.74
13	*Gemmobacter nectariphilus* DSM 15620^T^ (AUCM00000000)	95.3	74.69	22.11	95.3	74.82	22.17
14	*Gemmobacter serpentinus* HB-1^T^ (WCHR00000000)	96.6	77.38	21.15	96.6	77.42	21.22
15	*Gemmobacter straminiformis* CAM-8^T^ (JACLQD000000000)	95.9	75.20	22.16	95.9	75.12	22.24
16	*Gemmobacter tilapiae* KCTC 23310^T^ (BMYJ00000000)	97.3	73.41	23.00	97.3	73.63	23.15

**Table 3:** Descriptions of *Gemmobacter fulva* sp. nov.

Genus Name	*Gemmobacter*
Species name	*Gemmobacter fulva*
Species epithet	*fulva*
Species status	sp. nov.
Species etymology	ful′va. L. fem. adj. *fulva* tawny, yellowish brown, the color of the colonies
Description of the new taxon and diagnostic traits	Gram- negative, non-motile, aerobic, and rod-shaped. The colonies appeared yellow, convex, circular, and smooth, with entire edges, after being grown for two days at 30 °C on R2A agar. The temperatures range for growth is 4 to 37 °C, with an optimum 30 °C; the pH range for growth is pH 5–10, with an optimum at pH 7. Oxidase and catalase activities are present. In the API 20 NE test, it is positive for the urease, hydrolysis of aesculin, and gelatin and the assimilation of *N*-acetyl-glucosamine, d-glucose, d-mannitol, d-mannose, malate and maltose but negative for the rest. In the API ZYM test, it is positive for the esterase (C4), esterase lipase (C8), α-glucosidase, leucine arylamidase, and naphthol-AS-BI-phosphohydrolase but negative for the rest. NaCl concentration range for growth is 0–2%, with an optimum at 0%. The cells were found to be susceptible to amikacin (30 µg mL^−1^), ampicillin/sulbactam (1:1; µg mL^−1^), chloramphenicol (30 µg mL^−1^), erythromycin (30 µg mL^−1^), gentamicin (30 µg mL^−1^), kanamycin (30 µg mL^−1^), nalidixic acid (30 µg mL^−1^), rifampicin (30 µg mL^−1^), spectinomycin (25 µg mL^−1^), streptomycin (25 µg mL^−1^), teicoplanin (30 µg mL^−1^), tetracycline (30 µg mL^−1^), and vancomycin (30 µg mL^−1^) but resistant to lincomycin (15 µg mL^−1^).Major fatty acids are summed feature 8 (comprising C_18:1_ *ω*7*c* and/or C_18:1_ *ω*6*c*). The major polar lipids are phosphatidylethanolamine, phosphatidylglycerol, two unidentified glycolipids (GL1, GL2), and an unidentified aminophospholipid. The major respiratory quinone is Q-10.
Country of origin	South Korea
Region of origin	Sangju
Source of isolation	Anabaena culture
Sampling date (dd/mm/yyyy)	03/2020
Latitude (xx◦xx xx N/S)	–
Longitude (xx◦xx xx E/W)	–
16S rRNA gene accession nr.	MZ317889 (con5^T^) and MZ317888 (con4)
Genome accession number [RefSeq; EMBL;]	con5^T^, CP076361–CP076367; con4, JAHHWR000000000
Genome status	Complete for type strain, but incomplete for non-type strain
Genome size	4,677,627 (con5^T^), 4,576,129 (con4)
GC mol%	64.1% (con5^T^), 64.1% (con4)
Number of strains in study	2
Source of isolation of non-type strains	*Anabaena* culture
Designation of the Type strain	con5^T^
Strain collection numbers	KCTC 82247^T^ = JCM 34791^T^

## Data Availability

Not applicable.

## Supplementary Materials

The following are available online at https://www.mdpi.com/article/10.3390/microorganisms9122423/s1, Figure S1. Transmission electron micrograph of strains con5^T^ (**A**) and con4 (**B**) grown on R2A for 48 h at 30 °C. Bar,1 μm. Figure S2. Polar lipid profile for strains con5^T^ (**A**) and con4 (**B**). All the polar lipids were stained with 5% molybdatophosphoric acid (for total lipids), molybdenum blue (for phospholipids), ninhydrin (for amino lipids), and anisaldehyde/sulfuric acid (glycolipids). AL, unidentified aminolipid; APL, unidentified aminophospholipid; PE, phosphatidylethanolamine; PG, phosphatidylglycerol; PC, phosphatidylcholine; GL, unidentified glycolipid; PL, unidentified phospholipid. 1, first dimension of TLC; 2, second dimension of TLC. Figure S3. Phylogenomic tree based on genome sequences of the strains con5^T^ and con4 in the TYGS (https://tygs.dsmz.de/, accessed on 25 August 2021). The branch lengths are calculated in terms of GBDP distance formula d5. The numbers above branches are GBDP pseudo-bootstrap support values > 60% from 100 replications, with an average branch support of 95.1%. The tree was rooted at the midpoint [34]. Figure S4. Cluster analysis of the profiles obtained from ANI (**A**) and DDH values (**B**). Dendrograms were calculated on the basis of the average nucleotide identity scores based on the whole genomes using the unweighted pair group method with the arithmetic averages clustering algorithm (UPGMA). Figure S5. Graphic representation of circular genome plot of strain con5^T^. The circles from inside to outside represent GC skew ([G − C]/[G + C]) plot of the genome, GC content, CDS-reverse, CDS-forward, contigs, and position label. Table S1. General genome features of strains con5^T^ and con4. Table S2. Genomic feature of type strain con5^T^.
