# Supplementary material for: Genomic Insights into Denitrifying Methane-Oxidizing Bacteria Gemmobacter fulva sp. Nov., Isolated from an Anabaena Culture
Source: Microorganisms. 2021 Nov 24;9(12):2423. doi: 10.3390/microorganisms9122423 (PMC8709402; doi:10.3390/microorganisms9122423)
Supplement: Supplementary file 1 [file microorganisms-09-02423-s001.zip › microorganisms-1455750-supplementary.pdf]

microorganisms

MDPI

Supplementary Materials

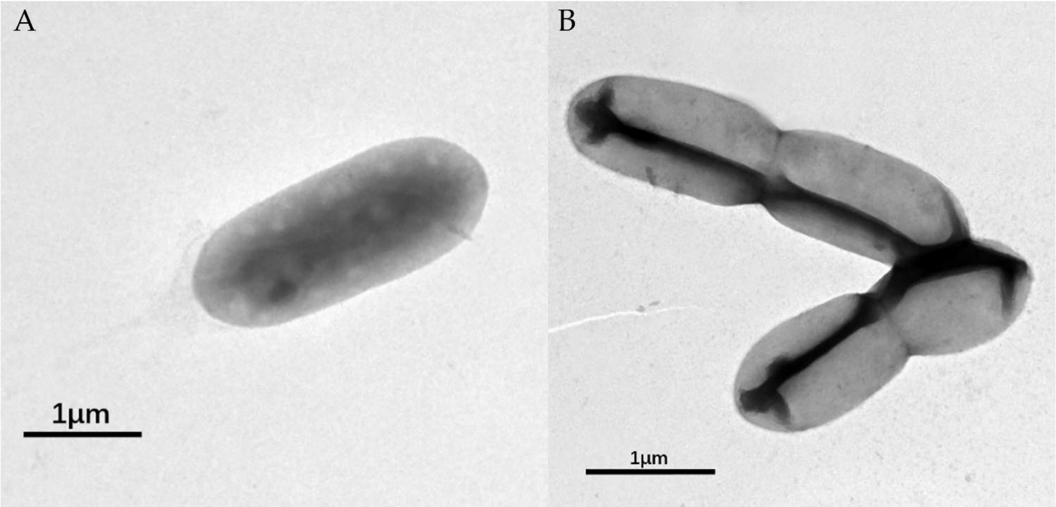

Figure S1. Transmission electron micrograph of strains con5^{T} (A) and con4 (B) grown on R2A for 48 h at 30 °C. Bar, 1 μm.

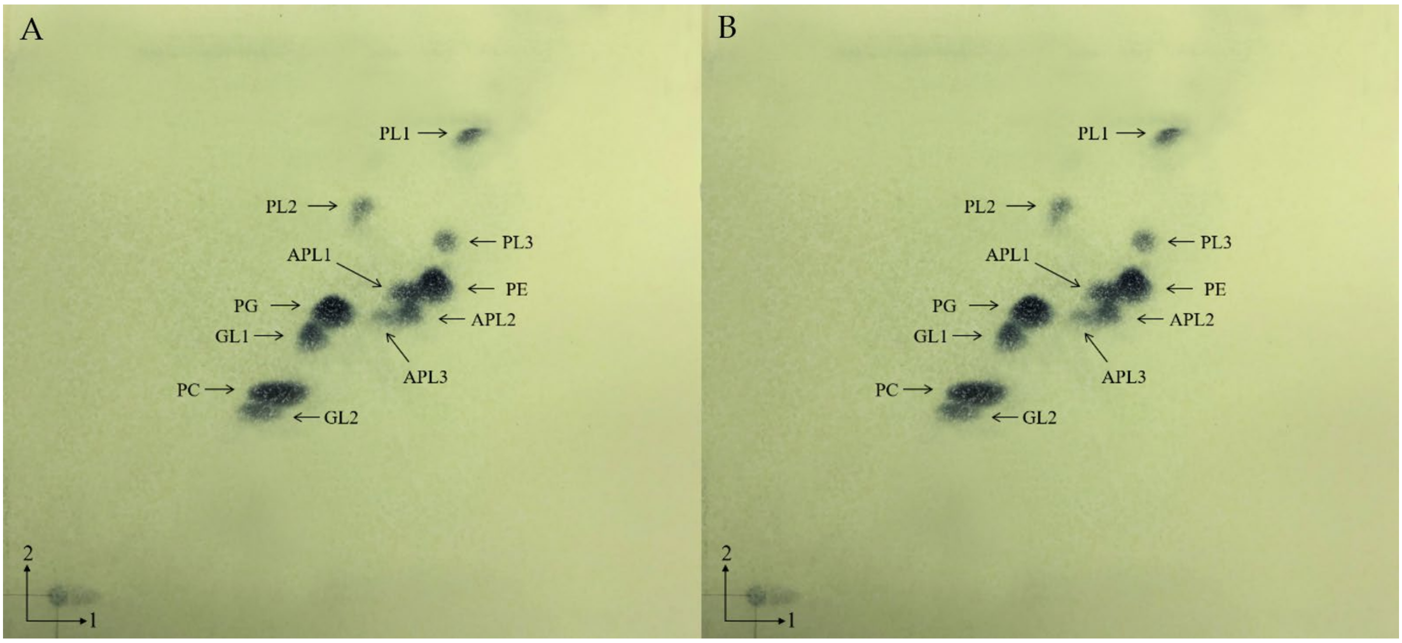

Figure S2. Polar lipid profile for strains con5^{T} (A) & con4 (B). All the polar lipids were stained with 5% molybdatophosphoric acid (for total lipids), molybdenum blue (for phospholipids), ninhydrin (for amino lipids), and anisaldehyde/sulfuric acid (glycolipids).AL, unidentified aminolipid; APL, unidentified aminophospholipid; PE, phosphatidylethanolamine; PG, phosphatidylglycerol; PC, phosphatidylcholine; GL, unidentified glycolipid; PL, unidentified phospholipid. 1, first dimension of TLC; 2, second dimension of TLC.

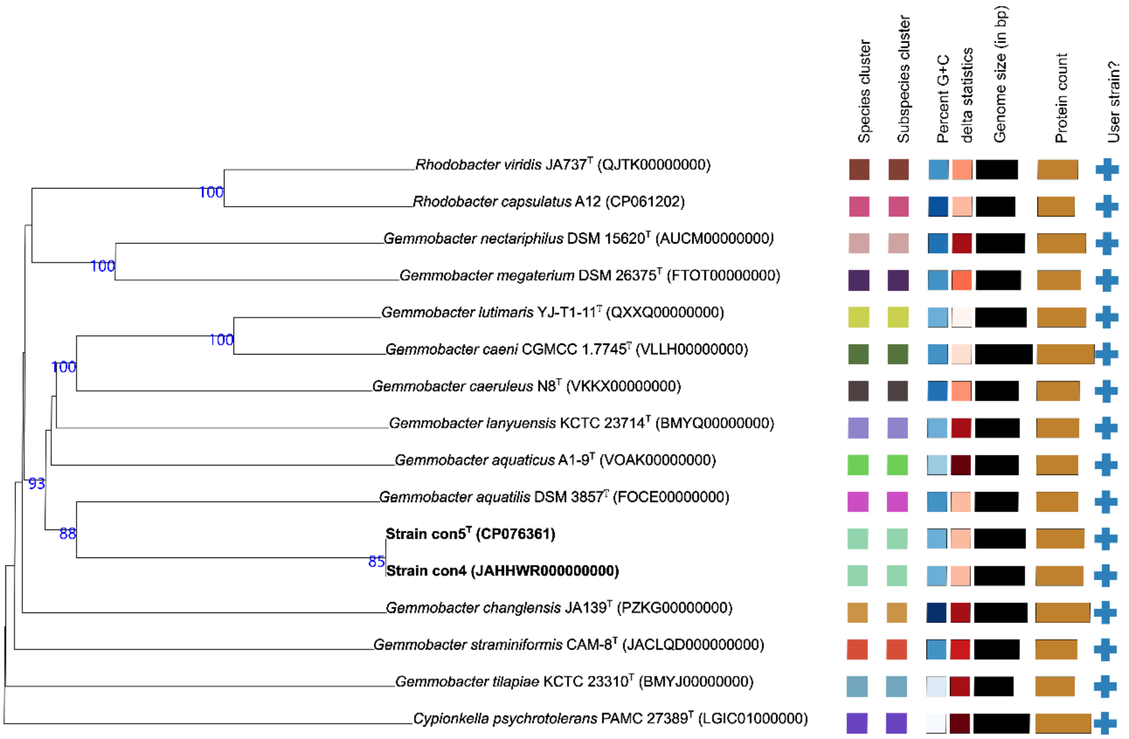

Figure S3. Phylogenomic tree based on genome sequences of the strains con5^T and con4 in the TYGS (https://tygs.dsmz.de/). The branch lengths are calculated in terms of GBDP distance formula d_5. The numbers above branches are GBDP pseudo-bootstrap support values>60 % from 100 replications, with an average branch support of 95.1 %. The tree was rooted at the midpoint [34].

A

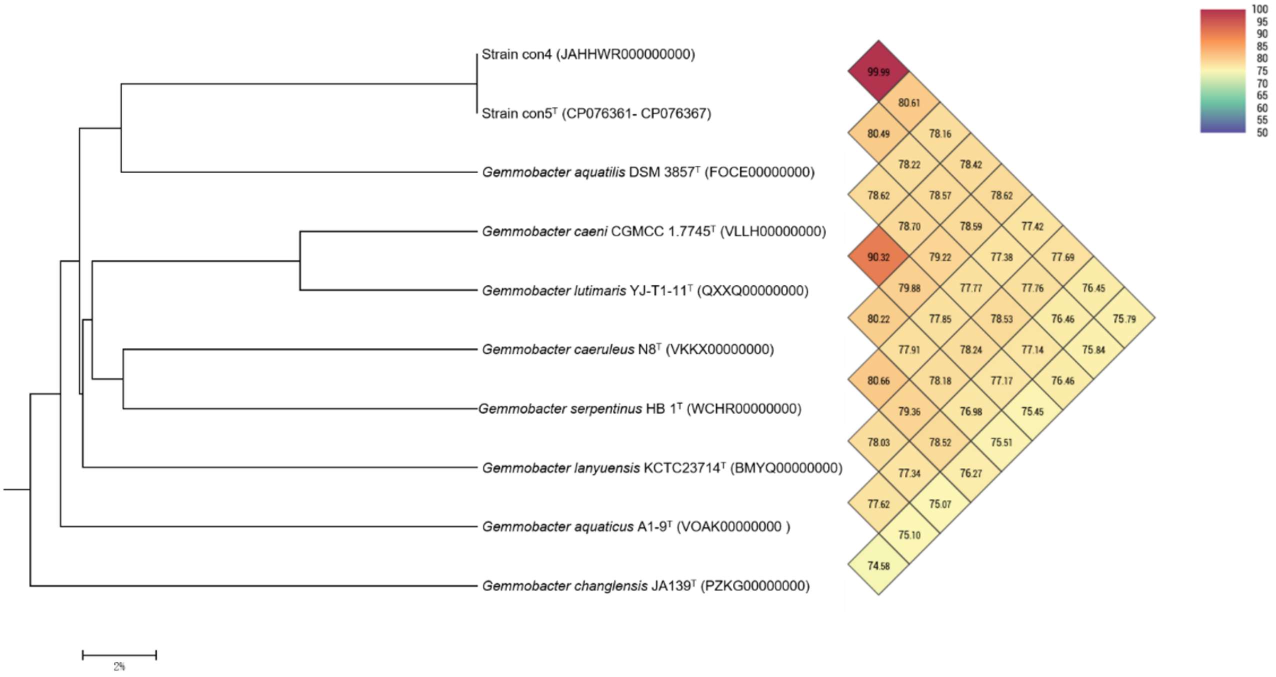

B

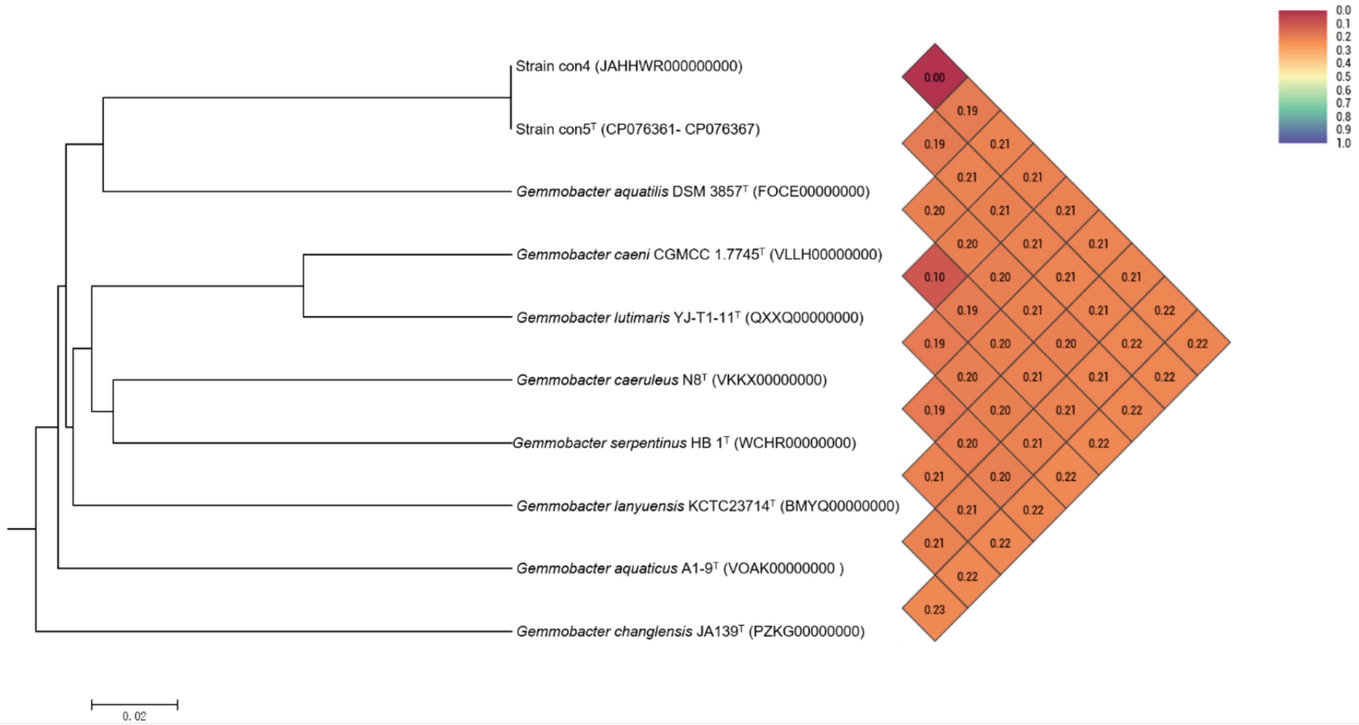

Figure S4. Cluster analysis of the profiles obtained from ANI (A) and DDH values (B). Dendrograms were calculated on the basis of the average nucleotide identity scores based on the whole genomes using the unweighted pair group method with the arithmetic averages clustering algorithm (UPGMA).

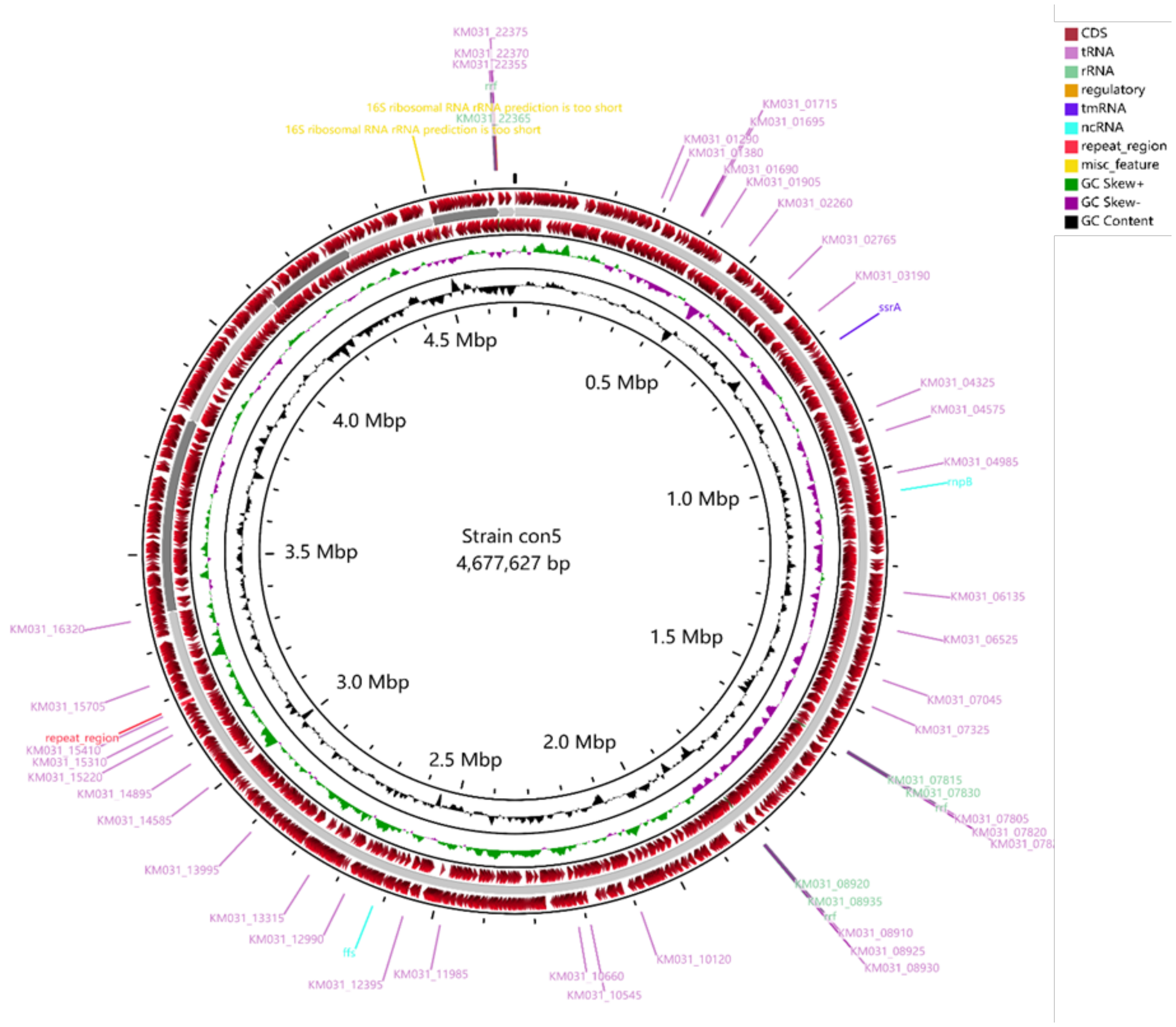

Figure S5. Graphic representation of circular genome plot of strain con5ᵀ. The circles from inside to outside represent GC skew ([G - C]/[G + C]) plot of the genome, GC content, CDS-reverse, CDS-forward, contigs and position label.

Table S1. General genome features of strains con5ᵀ and con4.

Attribute	con5ᵀ	con4
Genome size (bp)	4,677,627	4,576,129
Chromosome (bp)	3,376,470	-
Plasmids (bp)	1,301,157	-
Total genes	4,505	4,417
CDSs	4,444	4,364
rRNA genes (5S/16S/23S)	8 (3/2/3)	4 (1/2/1)
tRNA genes	50	46
ncRNA	2	3
Genome G + C content	64.1	64.1
GenBank Accession number	CP076361-CP076367	JAHHWR000000000

Table S2. Genomic feature of type strain con5^T.

Contig name	G+C content (%)	Length (bp)	Genes	tRNA	rRNA	tmRNA	ncRNA
Chromosome	64.4	3,376,470	3,269	47	6	1	2
Plasmid_1	64.5	425,522	430	-	-	-	-
Plasmid_2	64.3	310,690	291	-	-	-	-
Plasmid_3	60.8	196,089	180	-	-	-	-
Plasmid_4	62.6	193,372	175	-	-	-	-
Plasmid_5	62.8	141,504	135	3	2	-	-
Plasmid_6	59.5	33,980	29	-	-	-	-
Total	64.1	4,677,627	4,509	50	8	1	2
